# Exosomes from LPS-pretreated BMSCs treated periodontitis via improving oxidative stress

**DOI:** 10.1186/s13287-025-04860-y

**Published:** 2025-12-31

**Authors:** Chenyu Xu, Hanping Wang, Wenqi Dong, Wen Cheng, Yuran Su, Qiang Yang, Yue Wang, Yanhong Zhao

**Affiliations:** 1 Department of Orthodontics, Tianjin Medical University School and Hospital of Stomatology & Tianjin Key Laboratory of Oral Soft and Hard Tissues Restoration and Regeneration, No.12 Qixiangtai Road, Heping District, Tianjin, 300070 P. R. China; 2 Tianjin Medical University Institute of Stomatology, No.12 Qixiangtai Road, Heping District, Tianjin, 300070 P. R. China; 3 Department of Stomatology, Liangxiang Hospital of Beijing Fangshan District, Beijing, People’s Republic of China; 4 Department of Spine Surgery, Tianjin Hospital, Tianjin University, 406 Jiefang South Road, Tianjin, 300211 China; 5 Department of Orthopaedics, Beijing Friendship Hospital, Capital Medical University, Beijing, 100050 China

**Keywords:** Exosome, Periodontitis, Oxidative stress, Lipopolysaccharide, Bone marrow mesenchymal stem cell, Mitochondria

## Abstract

**Background:**

Research indicates that the occurrence of periodontitis is related to oxidative stress and mitochondrial dysfunction. Alleviating oxidative stress and mitochondrial dysfunction may be a promising treatment strategy for periodontitis. In this study, bone marrow mesenchymal stem cells (BMSCs) were pretreated with lipopolysaccharide (LPS), and their derived exosomes (LPS-BMSCs-Exo) were extracted. In vitro and in vivo experiments were conducted to study the therapeutic effects of alleviating oxidative stress, mitochondrial disorders, and periodontitis.

**Methods:**

BMSCs were pretreated with LPS, and LPS-BMSCs-Exo were extracted and identified via transmission electron microscopy (TEM), nanoparticle tracking analysis (NTA), and Western blotting. The biosafety of the exosomes was assessed through CCK-8, migration, and uptake assays. A cell oxidative stress model was established and treated with BMSCs-Exo or LPS-BMSCs-Exo, the following tests were performed: the effects of the two types of exosomes on the oxidative stress of periodontal ligament stem cells (PDLSCs) were determined, the mitochondrial state and the membrane potential were detected, the content of adenosine triphosphate (ATP) was determined, apoptosis was detected, and the effect of the exosomes on the osteogenic ability of the PDLSCs was detected. A periodontitis rat model was established, and PBS, BMSCs-Exo, and LPS-BMSCs-Exo were administered separately. Micro-CT, HE staining, Masson staining, immunohistochemistry, and ROS fluorescence staining were used to evaluate the therapeutic effect of each group on periodontitis in rats.

**Results:**

The proposed LPS-BMSCs-Exo exhibits characteristics similar to those of exosomes, can be successfully taken up and internalized by PDLSCs, and promotes the proliferation and migration of these cells. LPS-BMSCs-Exo can effectively improve the oxidative stress state, alleviate mitochondrial dysfunction in cells, increase membrane potential, enhance ATP content, reduce apoptosis, and improve the osteogenic ability of PDLSCs. Micro-CT data revealed that alveolar bone-related indicators were significantly increased after LPS-BMSCs-Exo treatment, which could reduce the degradation and inflammation of periodontal tissue in rats and alleviate their oxidative stress.

**Conclusion:**

LPS-BMSCs-Exo can significantly alleviate the oxidative stress and mitochondrial dysfunction caused by periodontitis in periodontal tissue, thereby reducing inflammation in periodontal tissue and alveolar bone resorption.

**Supplementary Information:**

The online version contains supplementary material available at 10.1186/s13287-025-04860-y.

## Introduction

 Periodontitis, a common oral infectious disease, can lead to irreversible periodontal attachment loss and alveolar bone resorption [1], serving as a primary cause of tooth loss in adults [2, 3] and posing a serious threat to human health. By 2019, the global number of severe periodontitis cases had reached 1.1 billion, affecting approximately 45%–50% of adults [3]. Recent studies indicate [4] that the pathogenesis of periodontal tissue destruction is associated with oxidative stress and mitochondrial dysfunction [5]. During the progression of periodontitis, periodontal pathogens such as Porphyromonas gingivalis release virulence factors including lipopolysaccharide. In response, host cells generate large amounts of reactive oxygen species (ROS) for antibacterial defense and signal transduction [6]. Excessive ROS accumulation triggers aberrant signaling cascades, such as the NLRP3/caspase-1, NF-κB/RANKL, and autophagy pathways. The overexpression of pro-inflammatory cytokines and proteolytic enzymes in host cells exacerbates inflammation [7, 8] and nonspecifically attacks macromolecules, leading to apoptosis, inhibition of osteogenesis, promotion of osteoclastogenesis, and ultimately alveolar bone resorption [9]. Clinical evidence shows that the structure and function of mitochondria are compromised in the periodontal tissues of periodontitis patients, which has also been validated in periodontitis model rats [10, 11]. The level of mitochondrial ROS in peripheral blood mononuclear cells from periodontitis patients is significantly higher than in periodontally healthy subjects. Furthermore, mtROS levels in peripheral blood mononuclear cells and lymphocytes decrease significantly after periodontal treatment [12, 13]. Mitochondria are highly sensitive to intra- and extracellular changes. Under stimuli such as hypoxia and peroxidation, mitochondria fail to adapt through quality control, leading to mitochondrial dysfunction—including impaired autophagy, disrupted biogenesis, and mtDNA mutations. This subsequently causes disturbances in energy metabolism and intracellular signal transduction, resulting in persistent inflammation, periodontal tissue destruction, and alveolar bone resorption [14–16]. In summary, during the progression of periodontitis, periodontal pathogens induce oxidative stress and trigger apoptosis via the mitochondrial pathway, ultimately leading to the destruction of periodontal supporting tissues and diminished repair capacity. This insight suggests that ameliorating mitochondrial function and mitigating oxidative stress could help control periodontitis.

In recent years, mesenchymal stem cells have been widely applied to control periodontal inflammation and repair tissue defects [17]. Bone marrow mesenchymal stem cells, adult stem cells residing in the bone marrow, possess multidifferentiation potential, self-renewal capacity, migration, and homing abilities, making them suitable seed cells for promoting periodontal tissue regeneration [18]. Moreover, BMSCs can inhibit ROS production by enhancing mitochondrial function and reducing mitochondrial damage [19], thereby modulating the oxidative stress process in periodontal cells and mitigating injury [20]. However, stem cell therapy has several limitations, including immune rejection of allogeneic cell transplantation, reduced stem cell viability, susceptibility to necrosis and apoptosis, and challenges in storage and transportation [21, 22]. Therefore, there is a need to identify suitable cell-free therapies, and exosome-based therapy has emerged as a promising alternative. Exosomes are cup-shaped vesicles, 30–150 nm in diameter, with a double-layered lipid membrane structure. They transport bioactive molecules to recipient cells via membrane fusion or endocytosis, thereby regulating gene expression, signal transduction, and physiological or pathological functions in target cells [23]. Previous studies have demonstrated that BMSCs-derived exosomes can promote periodontal tissue regeneration [24]. When exposed to specific external stimuli, the paracrine activity of BMSCs is significantly enhanced, and such “preconditioned” exosomes are believed to more effectively regulate target cells [25, 26]. Lipopolysaccharide, a key component of the gram-negative bacterial cell wall, plays a critical role in host–pathogen interactions and is a commonly used inflammatory inducer in studies of inflammatory diseases [27]. Pretreatment with low concentrations of LPS enables BMSCs to retain memory of danger signals or environmental stimuli for a period, resulting in improved therapeutic efficacy in modulating inflammation [28, 29]. Their derived exosomes also exhibit enhanced functionality.

**Fig. 1:**
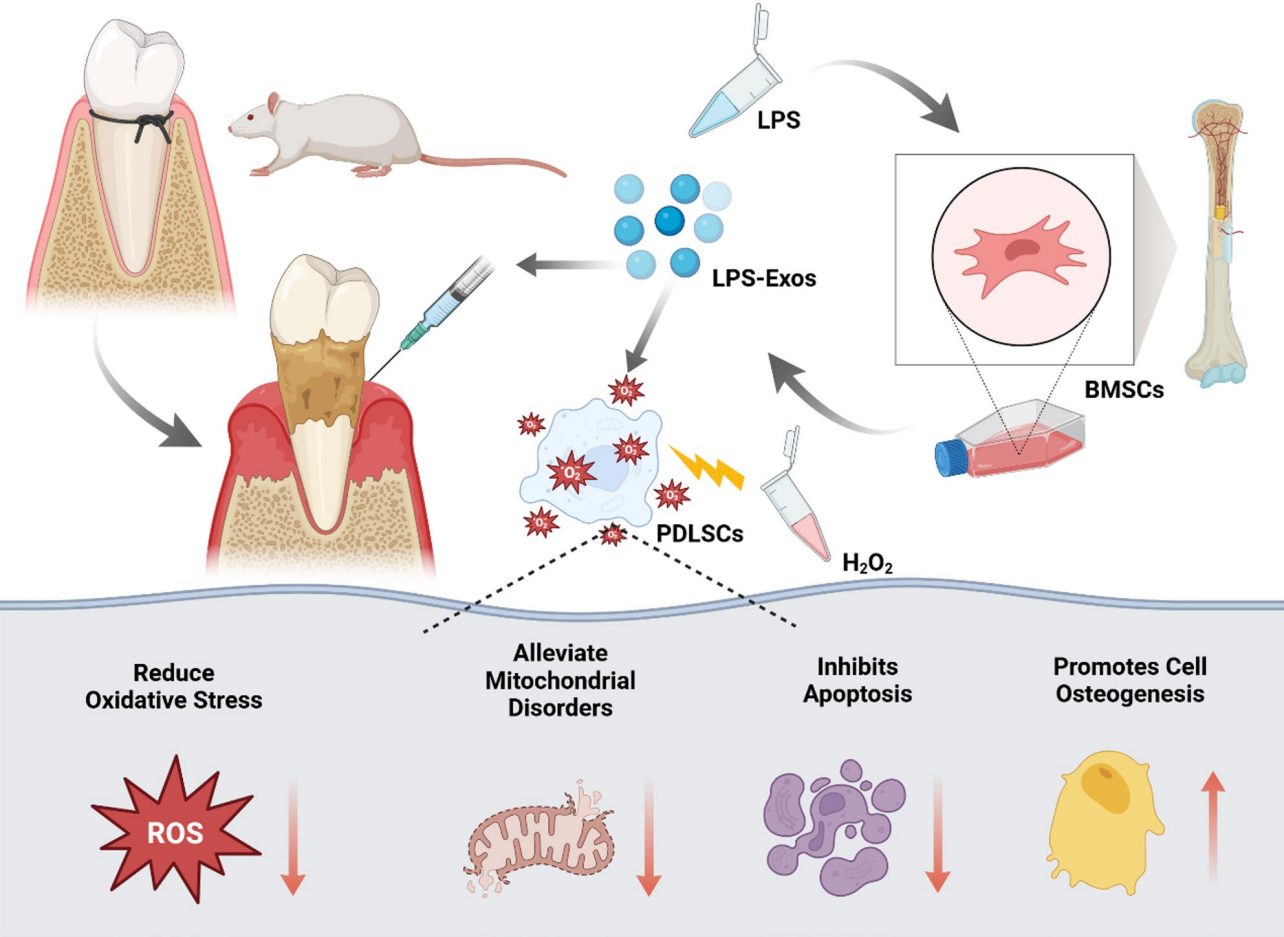
Schematic diagram of LPS-BMSCs-Exo in the treatment of periodontitis

Periodontal ligament stem cells, as key mesenchymal stem cells residing in periodontal tissues, play a central role in maintaining periodontal tissue homeostasis through their remarkable osteogenic capacity [30, 31]. Furthermore, during periodontal bone regeneration, PDLSCs not only directly participate in bone formation but also modulate the immune microenvironment and promote angiogenesis via paracrine signaling, thereby creating favorable conditions for bone regeneration [32]. Therefore, enhancing the osteogenic potential of PDLSCs is crucial for alveolar bone repair and regeneration. As shown in Fig. 1, this study aims to isolate exosomes from LPS-preconditioned bone marrow mesenchymal stem cells and investigate the role of these exosomes in regulating the oxidative stress status and mitochondrial function of PDLSCs. The ability of exosomes to alleviate inflammation and inhibit bone resorption in a rat model of periodontitis will be further validated through in vivo experiments, providing a novel experimental basis for controlling oxidative stress in the treatment of periodontitis.

## Materials and methods

### Cell culture

Rats bone marrow MSCs (mesenchymal stem cells derived from bone marrow of rats) were purchased from Wuhan Pricella Biotechnology Co., Ltd (Cat NO.: CP-R131). Cells were cultured in α-MEM culture medium (Gibco) supplemented with 10% fetal bovine serum (FBS, Gibco) and 1% Penicillin/Streptomycin/Amphotericin B, sterile solution (Solarbio) at 37 ℃ in a humidified atmosphere of 5% CO_2_. Human Periodontal Ligament Stem Cells (PDLSCs) were purchased from Wuhan Pricella Biotechnology Co., Ltd (Cat NO.: CP-H234). PDLSCs were cultured in Human periodontal ligament stem cells complete medium (containing FBS, growth additives, penicillin, Streptomycin, etc.) at 37 ℃ in a humidified atmosphere of 5% CO_2_. The BMSCs and PDLSCs isolated by the Pricella laboratory were identified by CD44 immunofluorescence, and the purity was more than 90%, and did not contain HIV-1, HBV, HCV, mycoplasma, bacteria, yeast and fungi.

### Animal experiment

Twenty-five Sprague-Dawley rats were used in this study. All experiments utilized male Sprague-Dawley (SD) rats aged 4–6 weeks. All the animal experiments were approved by the Institutional Animal Care and Use Committee of Yi Shengyuan Gene Technology (Tianjin) Co., Ltd. The animals were obtained from Beijing Huafukang Biological Technology Co., Ltd and were confirmed to be healthy, immunologically naive, non-genetically modified, and with no history of prior experimental use. Five rats were randomly designated as the normal control group, while the remaining twenty underwent periodontitis modeling and were subsequently randomized into four groups. An independent researcher blinded to subsequent procedures randomly assigned all animals to different groups using computer-generated numbers. A confidential code sheet linking each animal’s identifier to its group allocation was created and maintained solely by this individual. Briefly, rats were anaesthetized with 2% isoflurane (RWD) and then placed in a supine position with the mouth fixed and fully exposed. A 0.25 mm orthodontic ligature wire (Tiantian) was lightly abraded with sandpaper to enhance surface roughness, then ligated around the neck of the bilateral maxillary second molars and tightly secured.

After the procedure, all animals were monitored daily for recovery, mental state, and feeding activity. Bedding was refreshed regularly, and food and water were replenished as needed. The modeling period lasted for 3 weeks. Rats were excluded from the study if the ligature wire became dislodged during the modeling procedure. At the end of this period, the rats were anesthetized again, and the ligatures were removed to complete the modeling process.

Following the induction of periodontitis, the non-modeled rats were designated as the normal control group (Group 1). The remaining modeled rats were randomly allocated into four experimental groups (*n* = 5 per group): Group 2 (periodontitis group), Group 3 (periodontitis + PBS injection, PBS group), Group 4 (periodontitis + 40 µg/mL BMSCs-Exo injection, BMSCs-Exo group), and Group 5 (periodontitis + 40 µg/mL LPS-BMSCs-Exo injection, LPS-BMSCs-Exo group). Groups 2 to 5 received bilateral injections of 100 µL of their respective treatment solutions into the buccal and palatal periodontal pockets of the maxillary second molars. The injections were administered every other day for 2 weeks. After each injection, the feeding behavior and mental state of the rats were observed. After the 2-week treatment period, the rats were euthanized by CO₂ inhalation in a sealed chamber with a gradually increasing concentration from 30 to 70%.

### Cell proliferation assay

A Cell Counting Kit-8 (CCK-8) assay (Spark Jade) was used to evaluate the viability of the cells. The cells were seeded in 96-well plates at a concentration of 5 × 10^3^ cells/well. At specific time intervals, a dilution of 1:10 CCK-8 solution (10 µL) was added, and the plates were incubated for 1–4 h at 37 °C. The absorbance at 450 nm was measured with a microplate reader (Bio-Tek), which reflected the viability of the cells.

### Isolation and purification of exosomes

The BMSCs-Exo was extracted by gradient centrifugation from the supernatant of BMSC cultured in xeno-free mesenchymal stem cell medium for 48 h. For LPS-BMSCs-Exo, the culture supernatant was collected from BMSCs cultured in xeno-free mesenchymal stem cell medium (Applied Cell) containing 100 ng/mL LPS (Sigma) for 48 h. The collected culture medium was centrifuged at 300×*g* for 10 min, 2000×*g* for 10 min, and then 10,000×*g* for 30 min to remove cell debris and large vesicles. For exosome purification, the supernatant was ultracentrifuged at 120,000×*g* for 90 min at 4 °C to collect the pellet, which was then resuspended in 100 µL of PBS for subsequent studies. The concentration of the exosomes was detected via a BCA protein assay (Solarbio). For exosome addition, the culture medium of recipient cells was supplemented with purified exosomes at a concentration of 40 µg/mL, unless otherwise specified.

### Western blot

Exosomes were lysed via high-performance protein lysis buffer (RIPA: PMSF = 100: 1) (Spark Jade), 5 × SDS-PAGE protein loading buffer was added, the mixture was heated at 95 °C for 10 min, and 30 µg of exosomes was placed on an SDS‒PAGE gel (Epizyme) for electrophoresis. After several marker color bands near the molecular weight of the target protein were separated, electrophoresis was completed, and the proteins were transferred to a PVDF membrane (Millipore). The obtained membrane was blocked in 5% skim milk for 2 h at room temperature and then incubated at 4 °C for 12 h. The membranes were then incubated with secondary antibody for 2 h at room temperature. Finally, ECL (Epizyme) was used to wet the membrane with an automatic chemiluminescence image analyzer (Tanon), and the membrane was exposed and photographed.

### Electron microscopy and nanoparticle tracking analysis

Next, 20 µL of the exosome suspension was added to the center of the copper mesh and incubated at room temperature for 1 min. Then, 20 µL of 20 mM phosphotungstic acid negative staining solution was added. The pH was adjusted to 6–7 with sodium hydroxide, and the samples were negatively stained at room temperature for 1 min. The excess staining solution was removed, the copper mesh was shaken under an incandescent lamp for 10 min, and the samples were observed via transmission electron microscopy (TEM). During NTA detection, 10 µL of exosomes was taken and diluted 1000 times with PBS buffer after the membrane passed. The diluted exosome suspension was added to the sample room and scanned to calculate the size and concentration of the exosomes.

### Cell uptake experiment

The prepared Dio working solution (Beyotime) was mixed with the two groups of exosome suspensions at a 1:1 volume ratio and stained in the dark at room temperature for 30 min. The exosomes were washed three times with precooled PBS at 4 °C in an ultracentrifuge at 100,000×*g* for 90 min to remove the Dio staining solution that was not bound to the exosomes. Finally, the concentration of the exosomes was adjusted to 40 µg/mL with precooled PBS solution on ice. PDLSCs were seeded on slides, and Dio-labeled exosomes were cocultured with them for 24 h. After uptake was complete, phalloidin staining was performed. After washing, fixing, and permeabilization, the slides were incubated with the prepared phalloidin working solution, and staining was performed at 37 °C for 60 min in the dark. After the staining was completed, conventional washing, production, and sealing were performed. Finally, a laser confocal microscope was used in the dark for observation.

### Cell migration assays

Scratch experiment: PDLSCs were seeded in a 6-well plate at a density of 1 × 10^5^ cells/well. When the cell monolayer was observed to have fused, a 200 µL pipette tip was used to cross the cell surface and form a wound. The samples were then gently rinsed with PBS to remove excess cell debris. The exosome medium (without serum) was replaced, and wound healing was observed after 6 and 18 h. The wound healing area was then measured and quantified via ImageJ software. Transwell experiment: Hungried cells were seeded in each Transwell chamber at a density of 1 × 10^4^ cells/mL in serum-free medium. The chambers were placed in a 24-well plate, and the required liquid for each group was added to the corresponding well. After 24 h of normal culture, the chamber was removed, washed, fixed, and dried. After being stained with 0.1% crystal violet, the upper layer of the chamber was removed. Finally, the field of vision was randomly selected for image counting.

### ROS staining

The cells were washed three times with PBS and then added to a working solution of the fluorescent stain DCFH-DA (Beyotime). The samples were stained in the dark for 20 to 30 min, washed three times with serum-free medium, and observed under a fluorescence microscope. Representative images were randomly collected.

### SOD activity detection

The cells were routinely washed, and an appropriate volume of SOD sample preparation solution (Beyotime) was added to lyse the cells. After the preparation mixture became viscous, it was centrifuged for 5 min at 12,000×*g*, and the temperature was set to 4 °C. The protein concentration in the supernatant of the mixture was determined via a BCA kit. According to the proportions specified in the grouping reference manual, the samples obtained by centrifugation, the buffer solution, the mixed working solution of WST-8 and the enzyme, and the working solution for initiating the reaction were sequentially added to each group of orifice plates and thoroughly mixed. At room temperature, the entire reaction mixture was mixed and incubated for 20–30 min. The OD value at 450 nm was then measured, and the SOD level was calculated according to the provided instructions.

### JC-1 staining of mitochondria

At a ratio of 1:40:160, the JC-1 stock solution, buffer solution, and pure water were thoroughly mixed until the texture was uniform to obtain the JC-1 staining working solution (Beyotime). After the medium was changed, the cells were incubated with an appropriate volume of JC-1 working solution and incubated in the dark at 37 °C for 20–30 min. After the samples were washed with staining buffer, they were observed and photographed under a fluorescence microscope.

### Mito-tracker staining

Mito-Tracker Red CMXRos solution (Beyotime) was prepared as a stain storage solution at a concentration of 200 µmol/L in sterile DMSO, which was kept away from light and stored at − 20 °C. The storage solution was diluted to 200 nmol/L working solution, and the cells were stained with MitoTracker Red CMXRos working solution for 30 min in the dark. The medium was replaced with fresh medium, and the staining effect was observed under a microscope.

### Determination of ATP content

The cells were lysed with an appropriate volume of ATP lysis buffer (Beyotime), and the resulting mixture was centrifuged at 12,000×*g* for 5 min at 4 °C. The supernatant was used to determine the ATP content: an ATP detection working solution was quickly added to a nontransparent, healthy plate and allowed to stand for 5 min. A 1/5 volume of the supernatant or standard solution of the sample to be tested was added, the mixture was mixed well, and the RLU value was measured with a chemiluminescence instrument. The ATP content of each group was calculated.

### TUNEL staining

The cells were washed and then fixed. After washing again, the cells were permeabilized. Finally, the cells were washed three times with PBS. The 10 × TdT Enzyme and FITC-12-dUTP Labeling Mix (MeilunBio) was prepared in TUNEL detection solution at a ratio of 1:9 under dark conditions. The detection solution was added dropwise to each group of cells, and the sealing film was covered with a climbing film. After being incubated at 37 °C for 60 min, the cells were washed, mounted, and observed under a microscope, and photographs were taken for documentation.

### ALP staining

An alkaline phosphatase assay kit (Beyotime) and osteogenic medium were prepared according to the instructions. The cells were inoculated in a 24-well plate at a density of 1 × 10^5^ cells/well. After treatment, the osteogenic medium was replaced, and the medium was changed every 3 days. On the 7th day, the medium was discarded. After the cells were washed with PBS three times, they were stained with a well-conditioned staining solution for 1 h. The staining process was terminated by removing the staining solution, and the pure water was changed and observed.

### Alizarin red staining

After 14 days of osteogenic induction, the cells were washed once with PBS. The cells were fixed in paraformaldehyde for 20–30 min. After washing with PBS, an appropriate amount of alizarin red S staining working solution (Solarbio) was added to cover the cells evenly. After being incubated at room temperature for 30 min, the cells were thoroughly washed with distilled water and then observed and photographed under a microscope.

### Micro-CT

The maxillae were dissected for evaluation of alveolar bone resorption via high-resolution microcomputed tomography (micro-CT, SkyScan 1276, Bruker) with a source voltage of 70 kV and a source current of 200 µA. CTVox software and DataViewer software were used to reconstruct three-dimensional images and tomographic images of the alveolar bone. CTan software was used to measure and analyze the CEJ-ABC, BV/TV, BMD, and other indicators of maxillary alveolar bone in the rats.

### Histological staining

The maxillary specimens were gradually decalcified, embedded, sliced, and stained after fixation. They were decalcified with 10% EDTA, placed in a cool room at room temperature, and decalcified until the bone and tooth tissue of the sample became soft, allowing the needle to be easily inserted. The samples were dehydrated via a 75–95% gradient concentration of ethanol solution, treated with xylene for 2 h, waxed three times, and embedded before the wax block was completely solidified. The samples were then stored at -20 °C. After the wax block solidified completely, the tissue was cut into 4 μm thick slices via a microtome or slicing machine. After drying, the excess wax was shaken, and HE staining, Masson staining, and immunohistochemical staining were performed.

### HE staining and masson staining

Hematoxylin and eosin (H&E) staining was performed as follows: sections were stained with hematoxylin for 3 min, followed by rinsing in water, differentiation, and another rinse. Subsequently, sections were placed in a bluing solution for 1 min and rinsed again. This was followed by counterstaining with eosin Y for an appropriate duration. Finally, the sections were routinely dehydrated, cleared, and mounted with neutral gum.

For Masson’s trichrome staining, the protocol was carried out as follows: sections were first immersed in Masson’s A solution (hematoxylin) for 12 h and then rinsed. Next, a mixture of equal volumes of Masson’s B and C solutions was applied for 1 min, followed by a brief rinse in water. Differentiation was performed using Masson’s D solution, after which the sections were rinsed again. The sections were then stained with Masson’s E solution for 6 min. Excess liquid was carefully removed before applying Masson’s F solution for 1 min. Finally, the sections were differentiated for 2–10 s, followed by three rounds of conventional dehydration, clearing, and mounting.

### Immunohistochemical staining

The procedures for decalcification, embedding, and sectioning were consistent with those previously described. Following deparaffinization and hydration, endogenous peroxidase activity was quenched by incubating the sections in 3% H₂O₂ for 30 min in the dark. The sections were subsequently washed three times with PBS and subjected to antigen retrieval for 15 min, followed by cooling to room temperature. Subsequently, the sections were blocked with serum for 30 min at room temperature and then incubated with the primary antibody overnight at 4 °C. After washing three times, the sections were incubated with a secondary antibody for 1 h at room temperature. Color development was performed using a DAB chromogenic substrate, followed by counterstaining with hematoxylin. Finally, the sections were rinsed, differentiated, blued, dehydrated, and sealed.

### ROS fluorescence staining

Gingival tissues from each group were blotted dry on filter paper to remove surface moisture and subsequently snap-frozen in liquid nitrogen for 15 s. The samples were then stored at − 80 °C until further processing. For sectioning, the tissues were rapidly frozen and embedded, and serial sections were cut at a thickness of 8–10 μm. The sections were stored at − 20 °C and thawed to room temperature prior to staining. To reduce autofluorescence, the sections were treated with an autofluorescence quencher for 5 min and then washed in water for 10 min. Subsequently, the sections were incubated with Dihydroethidium (DHE) staining solution at 37 °C for 30 min in a dark environment, followed by counterstaining with DAPI for 10 min.

### Quantification and statistical analysis

All the experimental data in our study were collected at least three times. GraphPad Prism 8 (version 8.0.1) was used for statistical analysis. One-way ANOVA and an independent sample test were used to analyze the data of each group, and the variables are expressed as the average standard deviation (SD). *p* < 0.05 was considered statistically significant.

## Results

### LPS pretreatment of BMSCs

To determine the optimal concentration of LPS, combined with the concentration gradient used in reference [33], the CCK-8 method was used to detect the effects of different LPS concentrations on cell viability. Fig. S1 shows that cell viability was highest after treatment with 100 ng/mL LPS. Photographs taken under a light microscope revealed that the cells were in good condition (Fig. S2).

### Extraction and identification of exosomes

**Fig. 2:**
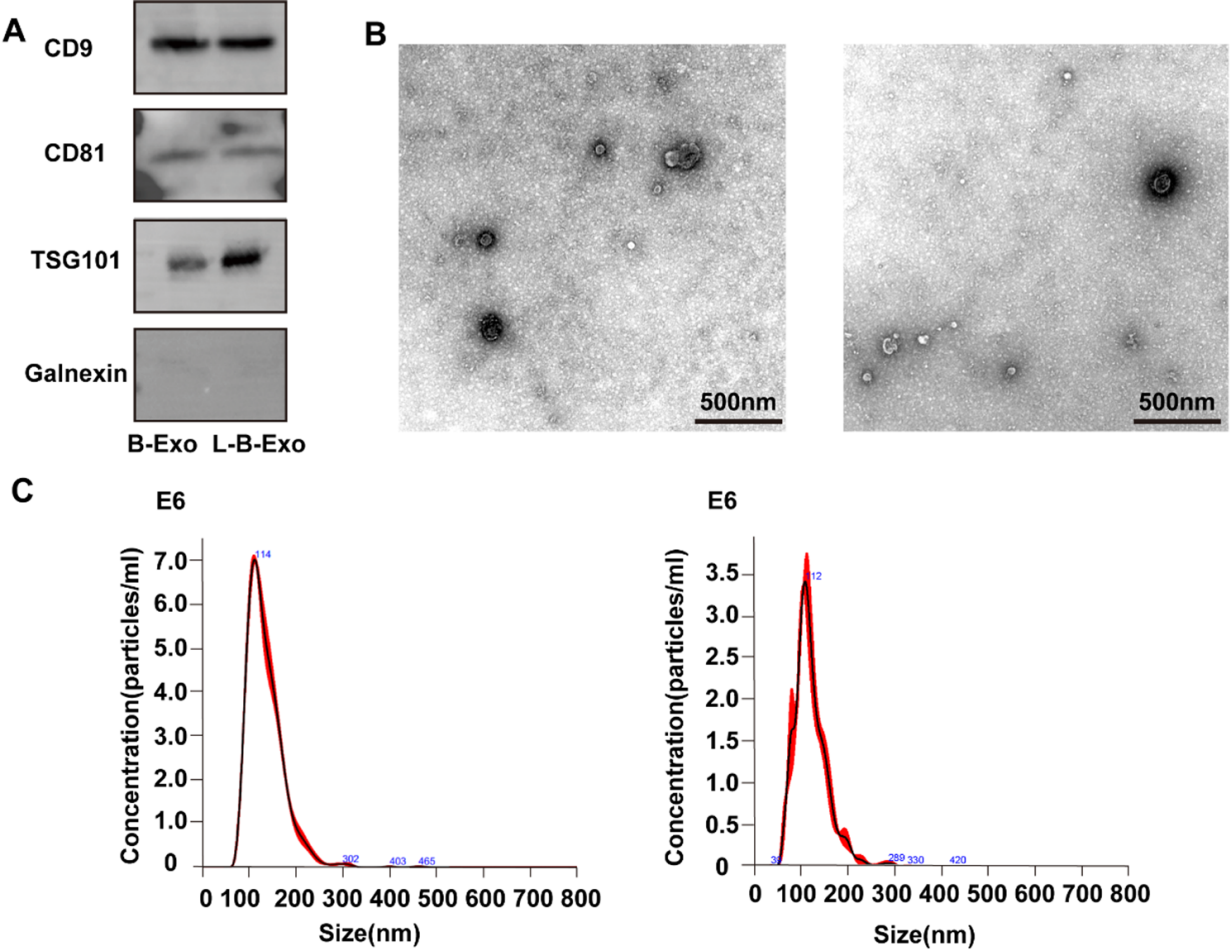
Characterisation results of exosomes. **A** Western blotting analysis of exosome surface marker protein expression. (Full-length blots are presented in Supplementary File 2). **B** TEM was used to identify the morphology of the two groups of exosomes. **C** NTA was used to determine the particle size of the exosomes in the two groups

Exosomes pretreated with/without LPS were extracted via ultracentrifugation and identified via Western blotting, TEM, and NTA. The western blot results revealed that both BMSCs-Exo and LPS-BMSCs-Exo could be positively correlated with specific exosome marker proteins, such as CD9, CD81, and TSG101 (Fig. 2A). TEM revealed that the morphology of BMSCs-Exo and LPS-BMSCs-Exo showed a typical round vesicle structure with a double-layer membrane structure (Fig. 2B), which was consistent with the literature [34]. The NTA results revealed that the diameters of the BMSCs-Exo and LPS-BMSCs-Exo were concentrated in the range of 100–200 nm. The peak particle size of the BMSCs-Exo group was 114 nm, and the peak particle size of the LPS-BMSCs-Exo group was 112 nm (Fig. 2C). These results confirmed that the exosomes were successfully extracted, and subsequent experiments were continued.

### Effects of BMSCs-Exo on the proliferation and migration of PDLSCs

**Fig. 3:**
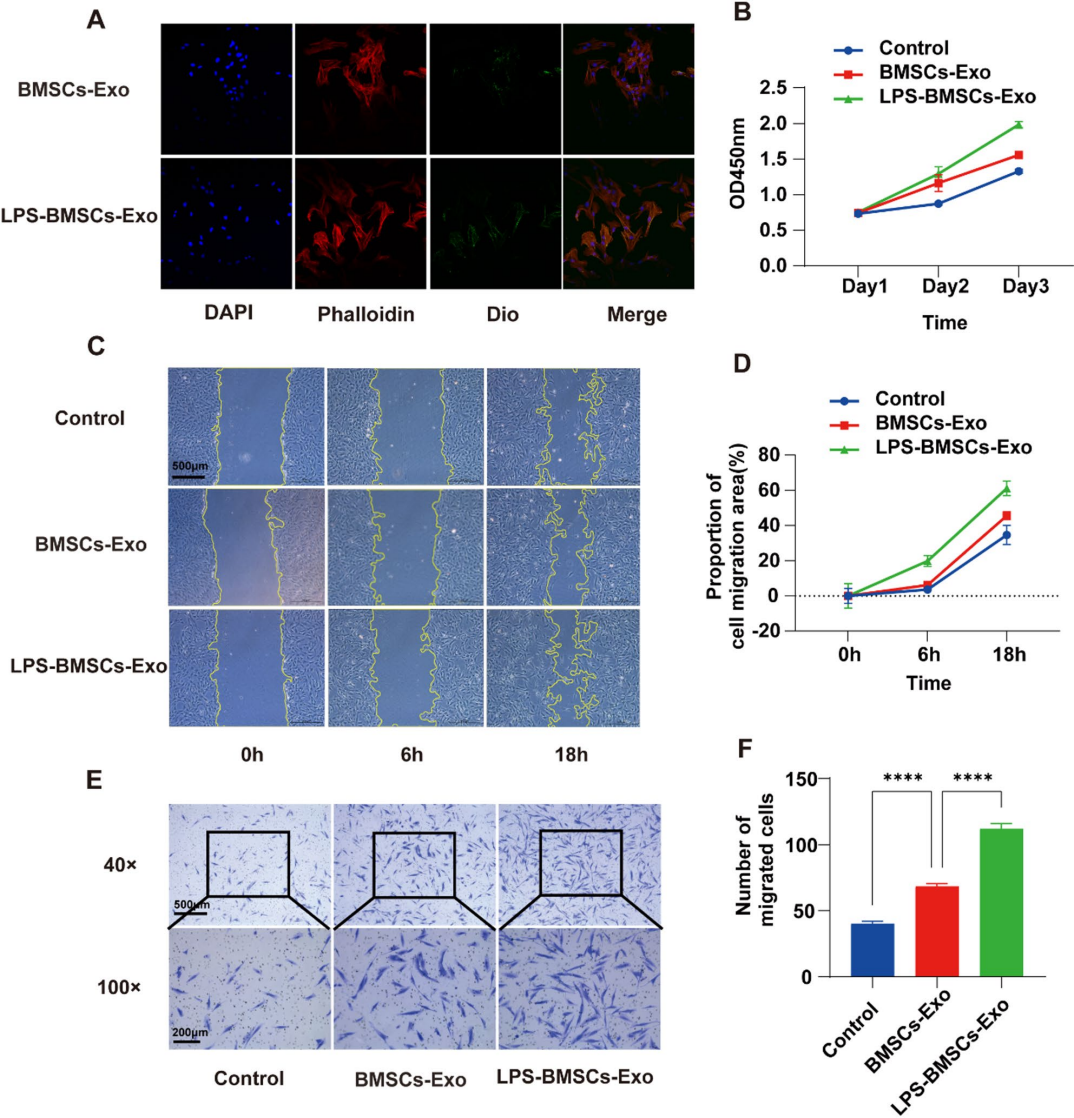
Biosafety of exosomes in vitro. **A** Uptake of exosomes by PDLSCs; **B** CCK-8 results showing the effect of exosomes on the proliferation of PDLSCs; **C** cell migration results of PDLSCs treated with BMSCs-Exo and LPS-BMSCs-Exo for 0 h, 6 h, and 18 h; **D** statistical results of the cell migration area; **E** results of the migration of PDLSCs treated with BMSCs-Exo and LPS-BMSCs-Exo for 24 h; **F** statistics of the number of migrated cells. **** *P* < 0.0001

PDLSCs were treated with two groups of exosomes (BMSCs-Exo or LPS-BMSCs-Exo) labeled with Dio at a concentration of 40 µg/mL for 24 h, and the uptake of the two groups of exosomes by PDLSCs was observed under a laser confocal microscope. As shown in Fig. 3A, PDLSCs can take up two groups of exosomes, and Dio-labeled exosomes are distributed around the nucleus of PDLSCs.

The CCK-8 method was used to assess the viability of PDLSCs at 24 h and 48 h after treatment with two groups of exosomes at a concentration of 40 µg/mL. The results are shown in Fig. 3B. As time progressed, both groups of exosomes promoted cell proliferation, and LPS-BMSCs-Exo had a more pronounced effect, which was statistically significant (Fig. S3). The impact of exosomes on the migration of PDLSCs to wounds was assessed via a scratch test. As shown in Fig. 3C and D, compared with that of the other two groups, the blank area of the LPS-BMSCs-Exo group decreased significantly as the treatment time increased (Fig. S4). The results of the Transwell assay revealed that the number of cells that migrated in the LPS-BMSCs-Exo group was considerably greater than that in the other two groups, with an increase in processing time (Fig. 3E and F).

### The ability of LPS-BMSCs-Exo to alleviate oxidative stress in cells

**Fig. 4:**
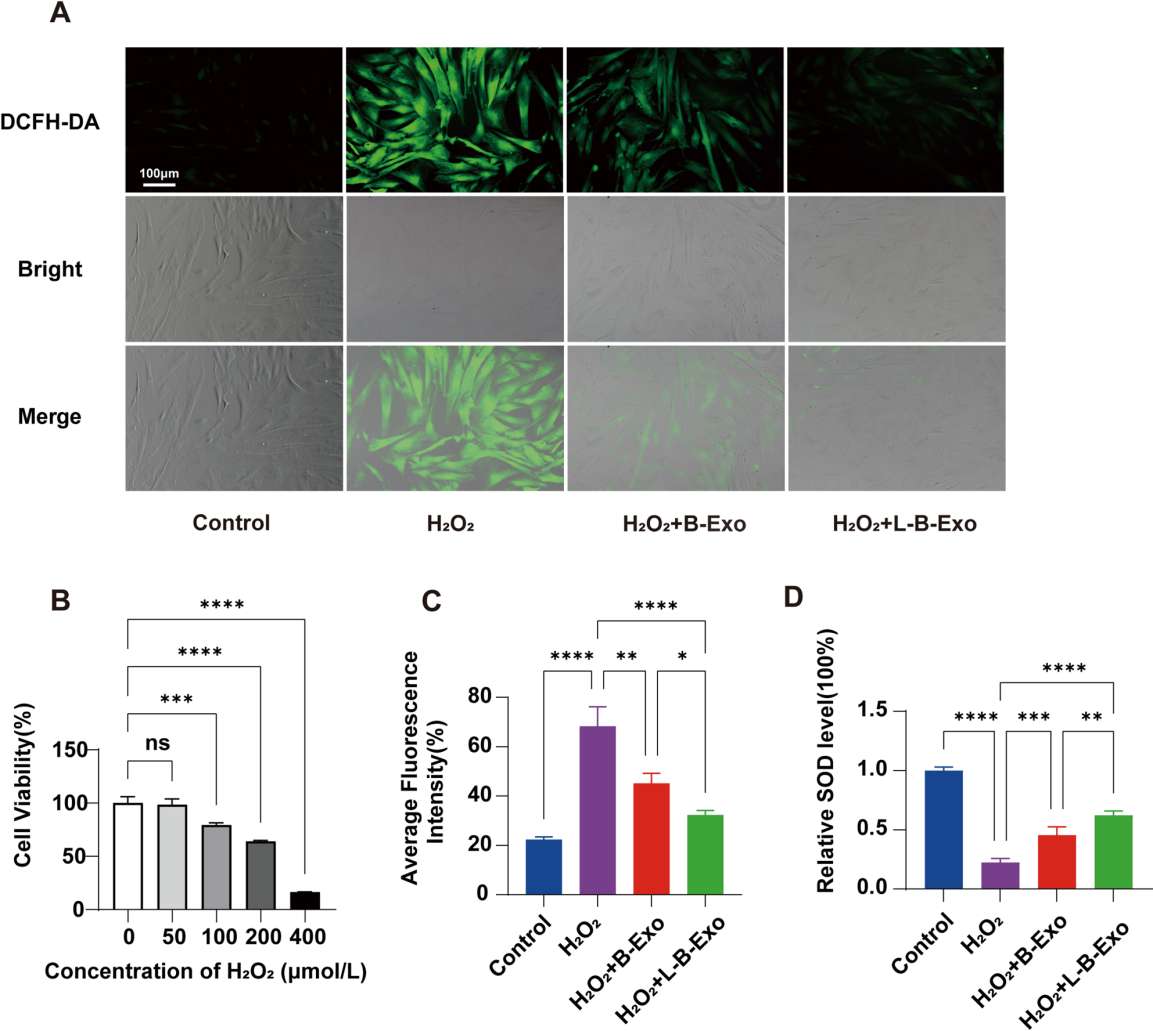
Antioxidant effect of exosomes. **A** Fluorescence staining of ROS production after PDLSC treatment in the control group, H_2_O_2_ group, H_2_O_2_ + BMSCs-Exo group, and H_2_O_2_ + LPS-BMSCs-Exo group. **B** Effects of different concentrations of H_2_O_2_ on the viability of PDLSC cells. **C** Statistical results of the fluorescence intensity of the four groups of cells in **A**. **D** SOD content changes in PDLSCs after treatment in each group. ** *P* < 0.01, *** *P* < 0.001, **** *P* < 0.0001

To determine the optimal concentration for the oxidative stress model, combined with the concentration gradient used in Reference [35], the CCK-8 method was used to assess changes in cell viability after PDLSCs were treated with various concentrations of H_2_O_2_. As shown in Fig. 4B, the effect of 50 μmol/L H_2_O_2_ on cell viability was negligible. After treatment with 100 μmol/L H_2_O_2_, the OD value of cell viability was significantly different from that of the blank group. Therefore, 100 μmol/L H_2_O_2_ treatment for 6 h was employed as a method to model cell oxidative stress.

To verify the effect of exosomes on H_2_O_2_-induced intracellular oxidative stress, this study evaluated the production of ROS and SOD activity in PDLSCs. The cells were treated with 100 μmol/L H_2_O_2_ for 6 h, and the medium was then discarded and replaced with complete medium containing or lacking exosomes. The cells were divided into four groups: a control group, an H_2_O_2_ group, an H_2_O_2_ + BMSCs-Exo group, and an H_2_O_2_ + LPS-BMSCs-Exo group. Figure 4A and C show the results of DCFH-DA cell staining. The fluorescence of the control group was weak, the green fluorescence of the H_2_O_2_ group was obvious, and a large amount of ROS was produced. The fluorescence of the H_2_O_2_ + BMSCs-Exo group was slightly weaker than that of the H_2_O_2_ group. The combination of H_2_O_2_ and LPS-BMSCs-Exo significantly inhibited the production of ROS.

As shown in Fig. 4D, compared with that of the control group, the SOD activity of the H_2_O_2_ group was impaired. Exosome treatment reversed the results of SOD damage. Compared with that in the BMSCs-Exo group, the recovery of SOD activity in the LPS-BMSCs-Exo group was more obvious. These results indicate that LPS-BMSCs-Exo can exert an antioxidant effect on PDLSCs under simulated oxidative stress.

### The ability of LPS-BMSCs-Exo to alleviate mitochondrial disorders in cells

**Fig. 5:**
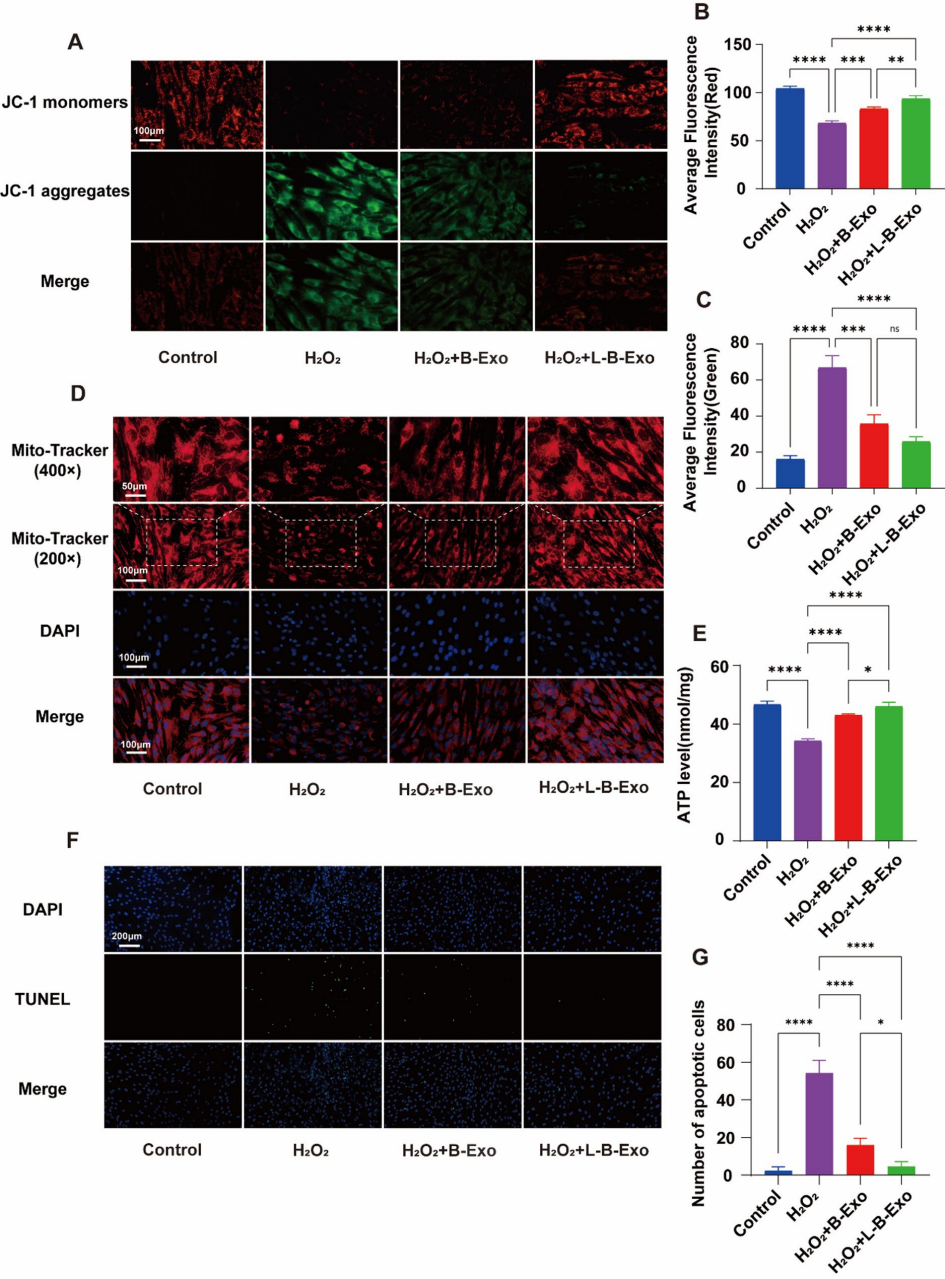
The function of exosomes on mitochondria. **A** JC-1 fluorescence staining results of PDLSCs in the control group, H_2_O_2_ group, H_2_O_2_ + BMSCs-Exo group and H_2_O_2_ + LPS-BMSCs-Exo group; **B** red fluorescence statistical results of JC-1 fluorescence staining results of the other groups compared with those of the H_2_O_2_ group; **C** green fluorescence statistical results of JC-1 fluorescence staining results of the other groups compared with those of the H_2_O_2_ group; **D** MitoTracker fluorescence staining results of PDLSCs in the four groups; **E** statistical results of ATP content in each group of cells after treatment; **F** TUNEL fluorescence staining results of PDLSCs in the four groups; **G** statistical results of the number of apoptotic cells in each group

JC-1 fluorescent probe staining can reflect the level of the mitochondrial membrane potential. The red fluorescence after staining indicates the production of polymers, suggesting that the mitochondrial membrane potential is high. The green fluorescence, on the other hand, represents the generation of monomers, indicating a low membrane potential. A decrease in the mitochondrial membrane potential increases the possibility of apoptosis. As shown in Fig. 5A–C, the red fluorescence of the control group and the LPS-BMSCs-Exo group was the strongest, and the green fluorescence was the weakest. The results revealed that the addition of H_2_O_2_ significantly decreased the mitochondrial membrane potential and that the membrane potential increased after BMSCs-Exo treatment. After LPS-BMSCs-Exo treatment, the membrane potential increased more than that in the BMSCs-Exo group. The results revealed that the addition of BMSCs-Exo and LPS-BMSCs-Exo promoted the recovery of mitochondrial function, and the effect of LPS-BMSCs-Exo was more significant.

As shown in Fig. 5D, MitoTracker fluorescence staining revealed that the fluorescence intensity of the control group and the LPS-BMSCs-Exo group was the strongest, followed by that of the BMSCs-Exo group, and the fluorescence intensity was the weakest in the H_2_O_2_ group. In addition, compared with those of the control group, the mitochondrial area and perimeter of the H_2_O_2_ group were significantly reduced, broken, and deformed, resulting in a collapsed mitochondrial network. The other two groups improved the morphology of the mitochondria; the mitochondrial network was more complete, and the perimeter was longer. The effect in the LPS-BMSCs-Exo group was greater than that in the other groups. As shown in Fig. 5E, H_2_O_2_ significantly reduced the intracellular ATP content, and LPS-BMSCs-Exo effectively alleviated ATP consumption, indicating that LPS-BMSCs-Exo dramatically improved the state and function of PDLSCs under oxidative stress.

As shown in Fig. 5F and G, the TUNEL results indicated that the apoptosis rate in the H_2_O_2_ group was greater than that in the control group. The apoptosis of PDLSCs treated with BMSCs-Exo and LPS-BMSCs-Exo improved, with the improvement induced by LPS-BMSCs-Exo being more pronounced.

### LPS-BMSCs-exo promotes the osteogenic function of PDLSCs

**Fig. 6:**
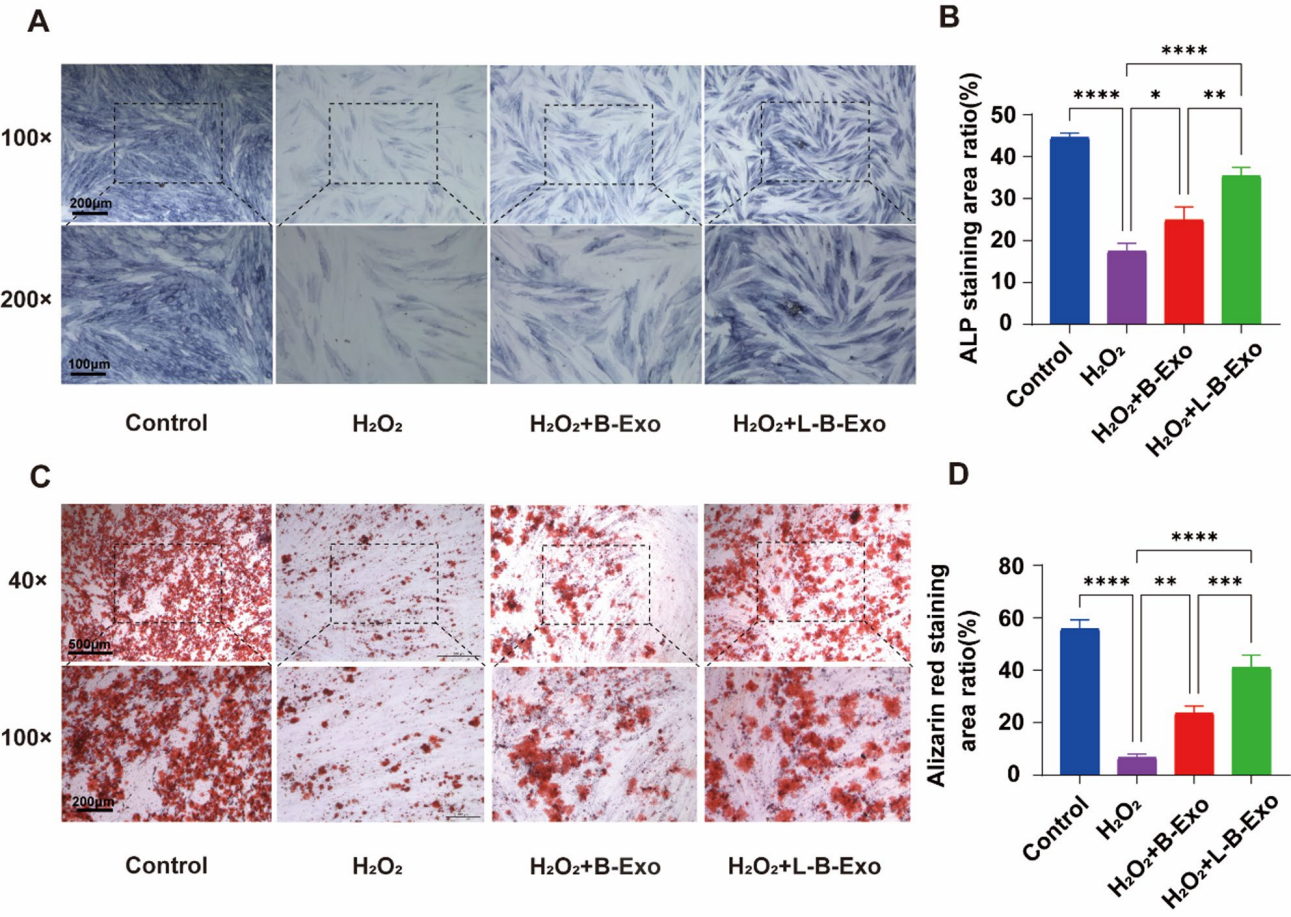
Exosomes can promote osteogenesis of PDLSCs. **A** ALP staining results of PDLSCs in the control group, H_2_O_2_ group, H_2_O_2_ + BMSCs-Exo group and H_2_O_2_ + LPS-BMSCs-Exo group; **B** Statistical results of the staining area of the four groups of cells in **A**; **C** Alizarin red staining results of PDLSCs in the four groups; **D** statistical results of the staining area of the four groups of cells in **C**

BCIP/NBT staining solution can be used to detect the production of ALP. ALP is an early indicator of the osteogenic differentiation of cells and was used to identify the early osteogenesis of PDLSCs in this study. Figure 6A shows the ALP activity after 7 days of osteogenic culture. The ALP activity of the H_2_O_2_ group was significantly lower than that of the control group, and the H_2_O_2_ group presented the lightest color. Compared with the H_2_O_2_ group, both the BMSCs-Exo and the LPS-BMSCs-Exo increased the ALP activity of the PDLSCs; however, the LPS-BMSCs-Exo had a greater promoting effect (Fig. 6B), indicating that the LPS-BMSCs-Exo promoted the early osteogenic differentiation of the cells. Alizarin red staining can be chelated with calcium ions to detect the osteogenic differentiation of PDLSCs effectively. As shown in Fig. 6C, the trend of the staining results was consistent with that of the ALP staining results, indicating that LPS-BMSCs-Exo effectively promoted the production of calcium ions in PDLSCs after 14 days of osteogenic culture (Fig. 6D).

### Therapeutic effects of LPS-BMSCs-Exo in periodontitis treatment

After the model ended, the rats woke up, and their mental state was poor; their water intake was also reduced. Three days later, the ligation wire had not fallen off, and the water intake had returned to normal. After 3 weeks of successful modeling, the ligation wire was removed, and the gums of the maxillary second molars of the rats were red and swollen. The periodontal pocket was deep and bled (Fig. S5).

**Fig. 7:**
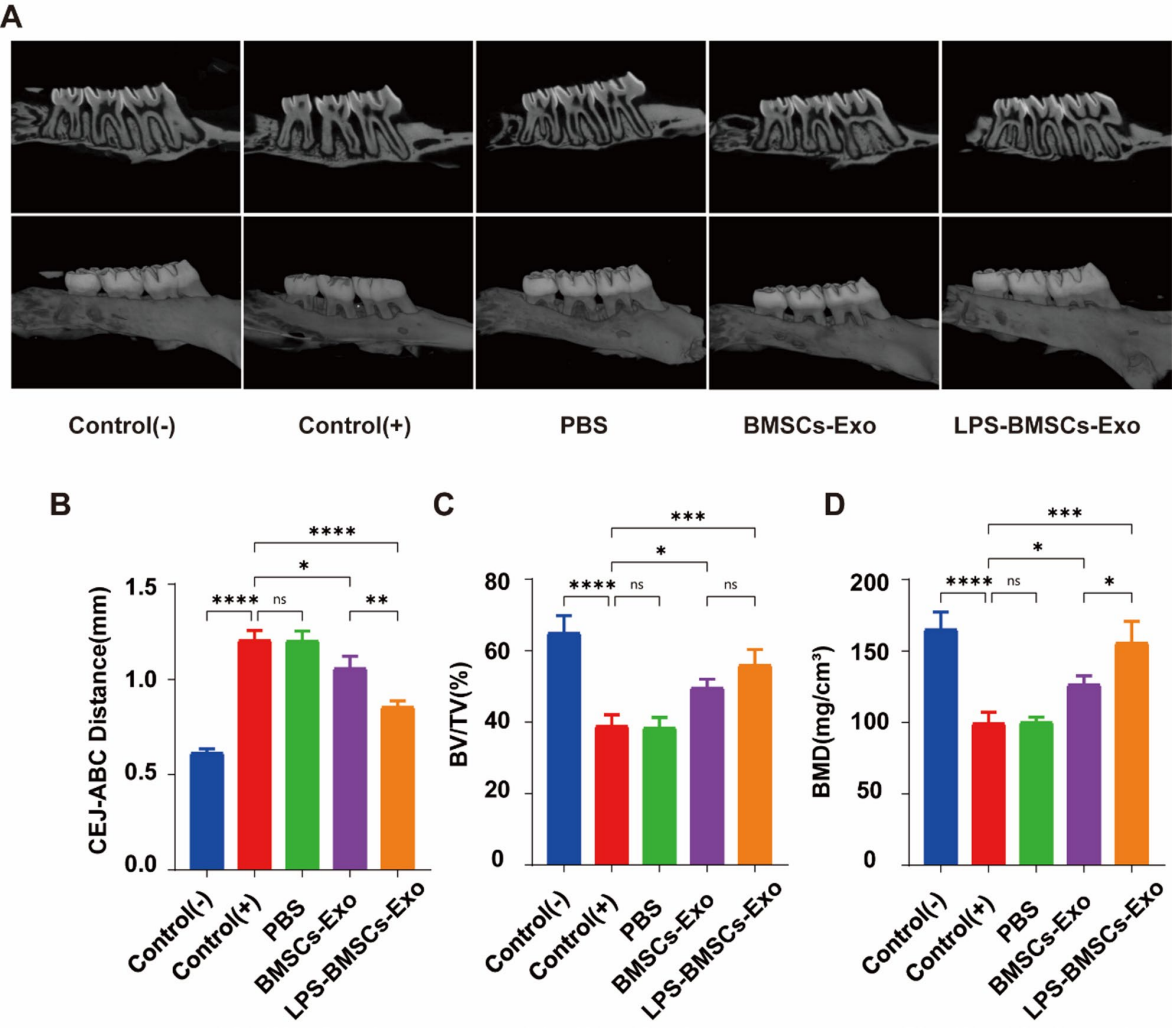
Micro-CT reconstruction and statistical results for each group. **A** Micro-CT reconstruction images; the upper row shows the sagittal section, and the lower row shows the 3D reconstruction effect. **B** CEJ-ABC, BMD, and BV/TV measurements and statistical results for each group and the other groups compared with those of the periodontitis group. *P < 0.05, *** *P* < 0.001, **** *P* < 0.0001

The results of micro-CT reconstruction are shown in Fig. 7A. Compared with that in the normal control group, the alveolar bone height of the maxillary second molars in the periodontitis group and PBS group was significantly lower, and the roots were exposed. The root bifurcation was involved, and the degree of alveolar bone resorption was significantly reduced in the rats treated with LPS-BMSCs-Exo. For the quantitative analysis of alveolar bone resorption in rats, the statistical results of CEJ-ABC, BMD, and BV/TV are shown in Fig. 7B and C, and Fig. 7D. The statistical results of the CEJ verified the results of the above alveolar bone height comparison. The BMD and BV/TV index reflect the alveolar bone density around the maxillary second molar to a certain extent; the bone mineral density of the LPS-BMSCs-Exo group was closest to that of the normal control group.

### Histological staining and analysis

**Fig. 8:**
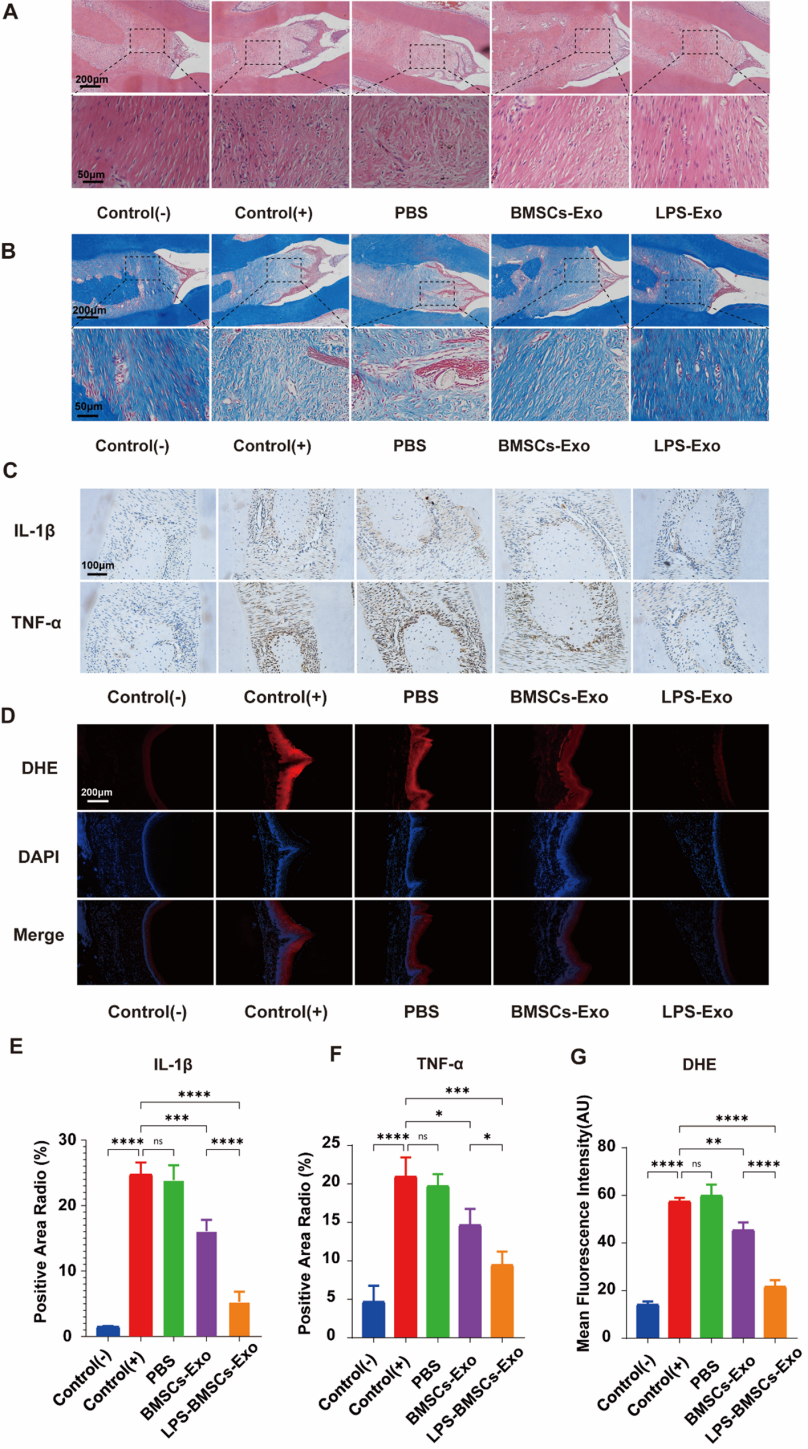
Histological staining results. **A** HE staining; the upper row is 100× field of view; scale bar: 200 μm. The lower row is a 400 × field of view, scale: 50 μm. **B** Masson staining; the upper row is a 100 × field of view, scale: 200 μm; the lower row is a 400 × field of view, scale: 50 μm. **C** Immunohistochemical staining results, scale: 100 μm. **D** ROS fluorescence staining results, scale: 200 μm. **E** Statistical results of IL-1β immunohistochemical staining. **F** TNF-α immunohistochemical staining. **G** Statistical Results of DHE Immunofluorescence Staining

HE and Masson staining (Fig. 8A and B) revealed that the periodontal tissue was significantly damaged in both the periodontitis group and the PBS group—the degeneration and degradation of elastic fibers and collagen fibers in periodontal tissue caused by inflammation. The arrangement of the fibers was disordered and sparse, and the height of the alveolar bone was significantly reduced. The results showed that the periodontitis model was successful, the periodontal tissue was damaged, and PBS treatment for periodontitis was ineffective. The degree of degradation and inflammation of periodontal tissue in the BMSCs-Exo group was lower than that in the periodontitis group, and the BMSCs-Exo slightly improved periodontal inflammation. The LPS-BMSCs-Exo had a favorable therapeutic effect on periodontal tissue repair, characterized by reduced inflammatory cell infiltration, denser elastic and collagen fiber tissue, and a more regular arrangement. The therapeutic effect of LPS-BMSCs-Exo was most significant.

The expression of the inflammatory cytokines IL-1β and TNF-α in periodontal tissue was detected via immunohistochemical staining (Fig. 8C). Compared with those in the normal control group, the expression levels of IL-1β and TNF-α antibodies in the periodontal tissues of the periodontitis group were significantly greater. The expression levels of IL-1β and TNF-α in the BMSCs-Exo group decreased slightly, indicating that BMSCs-Exo have a specific therapeutic effect on periodontitis; however, the effect is not yet apparent. The levels of IL-1β and TNF-α antibodies in the LPS-BMSCs-Exo group were significantly decreased, indicating that LPS-BMSCs-Exo effectively controlled periodontal tissue inflammation (Fig. 8E and F).

As shown in Fig. 8D, the results of ROS-dependent dihydroethidium (DHE) fluorescence staining indicated that the fluorescence signals of the normal control group and the LPS-BMSCs-Exo treatment group were lower than those of the control group. In contrast, the fluorescence signals of the periodontitis group and the PBS group were greater. The fluorescence of the BMSCs-Exo was between that of the periodontitis group and that of the LPS-BMSCs-Exo group, indicating that LPS-BMSCs-Exo can effectively alleviate oxidative stress in the treatment of periodontitis (Fig. 8G).

### Safety assessment of LPS-BMSCs-Exo

The rats in the LPS-BMSCs-Exo group were in good condition throughout the treatment period. After treatment, the rats were sacrificed, and H&E staining was performed to evaluate the in vivo toxicity of the LPS-BMSCs-Exo (Fig. S6). The results revealed that there was no damage to the main organs, such as the heart, liver, spleen, lungs, and kidneys. In detail, the structure and morphology of the myocardial fiber tissue were standard, and no inflammatory cell infiltration was observed. The liver cells were arranged neatly in the form of a liver plate, forming a regular hepatic lobule structure, and no noticeable pathological changes were observed. The spleen sections revealed that the white pulp was dark blue or blue purple and that the red pulp was red or pink, with a clear structure and no abnormalities. The alveolar structure is clear: the alveolar wall is composed of a single layer of alveolar epithelial cells. The alveolar epithelial cells were arranged neatly without abnormal hyperplasia or shedding, and the alveolar septum showed no abnormal thickening or fibrosis. The alveolar space was filled with air, and there were no abnormal phenomena, such as inflammatory cell infiltration, bleeding, or exudation. There was no inflammatory cell infiltration, bleeding, exudate, or other abnormal phenomena in the kidney tissue. Glomerular cells and renal tubular epithelial cells were arranged neatly, with no abnormal proliferation, shedding, or necrosis. LPS-BMSCs-Exo exhibit good biosafety and are nontoxic to organisms.

## Discussion

Periodontitis is a common chronic infectious disease in the oral cavity. In its middle to advanced stages, the disease is often accompanied by progressive and irreversible destruction of periodontal tissues [36, 37]. Periodontitis is typically associated with oxidative stress, a complex biological process characterized by excessive production of ROS, which disrupts the body’s redox homeostasis and induces oxidative damage [38]. Under normal conditions, low concentrations of ROS produced by cells facilitate cellular signaling and antibacterial responses, thereby maintaining normal cellular vitality and function. However, when ROS are overproduced, the balance between the pro-oxidant and antioxidant systems is disrupted, leading to oxidative stress [39]. In a state of oxidative stress, the body’s capacity to eliminate substances such as ROS is exceeded [40]. As a result, all metabolic activities can be compromised, and ROS contribute to tissue oxidative damage through various mechanisms, including DNA damage, protein oxidation, and lipid peroxidation. In this study, a rat periodontitis model was established, and DHE immunofluorescence staining of gingival tissues revealed significantly elevated ROS levels in the gingiva of periodontitis rats, confirming the occurrence of oxidative stress during the development of periodontitis. Additionally, a cellular oxidative stress model was constructed using hydrogen peroxide. Results showed a significant increase in intracellular ROS levels and a marked reduction in SOD activity in periodontal ligament stem cells, indicating the successful establishment of the oxidative stress model.

Mitochondria are essential organelles in eukaryotic cells, responsible for energy production, maintenance of fundamental cellular functions, and fine regulation of cell survival, death, and metabolism [41]. Cells rely on ATP generated by mitochondria to drive biochemical reactions, control transmembrane transport of substances, and regulate cell growth and the cell cycle [42]. Under physiological conditions, basic activities of periodontal tissue cells—such as proliferation, differentiation, and mineralization—are dependent on mitochondrial function. When challenged by periodontal pathogens, excessively produced reactive oxygen species (ROS) target mitochondria [43, 44], leading to structural damage, decreased membrane potential, and reduced mitochondrial DNA copy number. These alterations result in diminished ATP synthesis and the release of pro-apoptotic factors [45, 46] ultimately triggering inflammatory responses and even apoptosis, which contribute to the destruction of periodontal tissues [47].

In this study, further mitochondrial function and cellular activity assays performed on the oxidative stress cell model revealed that oxidative stress induced a decrease in mitochondrial membrane potential and functional impairment in periodontal ligament stem cells, accompanied by increased apoptosis. Additionally, ALP and Alizarin Red staining results indicated a significant suppression of osteogenic differentiation capacity. These findings suggest that oxidative stress may impair the osteogenic differentiation of periodontal ligament stem cells by disrupting mitochondrial function, thereby affecting the expression of signaling pathways related to osteogenesis.

Recent studies have shown that mesenchymal stem cell-derived exosomes can alleviate inflammation and synergistically promote bone regeneration by modulating the IL-17/RANKL signaling axis. Tang Wen et al. [48, 49] found that BMSCs-Exo inhibited the IL-17 signaling pathway to resist ferroptosis and promote functional recovery after spinal cord injury. Fu Yanxia et al. [49] reported that HUMSC-Exo restored immune balance in rheumatoid arthritis by regulating Th1/Th17 and Treg cells, accompanied by decreased IL-17 levels and increased TGF-β/IL-10 levels. Some researchers have reported that when MSCs are pretreated with certain disease-related derivatives (such as interleukins or LPS), their derived exosomes exhibit enhanced efficacy in tissue repair [47, 50]. Nakao Y et al. [51] demonstrated that exosomes from TNF-α-pretreated gingival mesenchymal stem cells carried miR-1260b and inhibited osteoclast differentiation of periodontal ligament stem cells via the Wnt5a-RANKL pathway. Among various pretreatment methods, LPS—one of the most common virulence factors derived from the periodontal pathogen *Porphyromonas gingivalis*, is enriched in the gingival tissues and peripheral blood of periodontitis patients and is associated with the severity of periodontal dysfunction [50, 52]. Previous studies have confirmed that low concentrations of LPS can enhance the paracrine activity and immunomodulatory capacity of BMSCs [53], promote exosome secretion, and improve exosomal function. In this study, by comparing the effects of BMSCs-Exo and LPS-BMSCs-Exo on PDLSCs, we found that LPS pretreatment enhanced the functional efficacy of BMSCs-Exo [52]. This study provides compelling evidence through in vivo and in vitro experiments, demonstrating the potential therapeutic value of LPS-BMSCs-Exo in periodontitis. Both BMSCs-Exo and LPS-BMSCs-Exo were able to alleviate oxidative stress and mitochondrial dysfunction, thereby reducing PDLSCs apoptosis and promoting their osteogenic differentiation, with LPS-BMSCs-Exo exhibiting more pronounced improvements. In a rat periodontitis model, LPS-BMSCs-Exo demonstrated superior therapeutic effects compared to BMSCs-Exo, significantly reducing tissue inflammation and oxidative stress levels, while inhibiting alveolar bone resorption.

Although the specific components in LPS-BMSCs-Exo responsible for regulating PDLSCs function remain unclear, we plan to systematically analyze their contents using proteomics and metabolomics in the future, aiming to identify specific molecules that may function through the “mitochondria-oxidative stress-osteogenesis” signaling axis, thereby further elucidating their mechanism of action. Based on existing studies, we hypothesize that exosomes may synergistically alleviate oxidative stress and promote osteogenesis through the following mechanisms, with their core function centered on the regulation and restoration of mitochondrial function. On one hand, exosomes can activate key signaling pathways to systematically ameliorate mitochondrial homeostasis. For instance, Zhang et al. [54] reported that hypoxia-induced hucMSCs-Exos enhance mitochondrial function by upregulating the SIRT3/PGC-1α pathway; Zhuang et al. [55] found that BMSCs-Exo alleviates oxidative stress and mitochondrial damage while restoring cellular energy metabolism through activation of the AMPK/PGC-1α pathway. On the other hand, exosomes can serve as carriers of mitochondrial genetic material, directly transferring mtDNA to damaged mesenchymal stem cells, thereby reconstructing mitochondrial function at the genetic level and restoring their osteogenic differentiation capacity [56]. Furthermore, studies indicate that mitochondrial transfer is an effective strategy for regulating oxidative stress. For example, MSCs can deliver functional mitochondria to damaged cells via extracellular vesicles, reversing metabolic dysfunction and sustaining ATP production [57].

In summary, exosomes likely employ a dual strategy of “signaling regulation” and “material delivery” to correct mitochondrial dysfunction while providing the energy foundation for osteogenesis. By improving cellular metabolism and reducing oxidative stress, they synergistically promote tissue repair. Moving forward, we will build upon these findings to further elucidate the detailed mechanisms by which LPS-BMSCs-Exo treats periodontitis, validate its potential as a cell-free therapeutic strategy, and provide a scientific basis for clinical translation.

Our study has several limitations that should be considered. First, the selection of the LPS pretreatment concentration was primarily based on a preliminary assessment of BMSCs cell viability. However, the functional effects of the derived exosomes were not evaluated; screening their biological activities across multiple concentration gradients would allow for a more precise determination of the optimal concentration, thereby providing deeper mechanistic insights. Second, while this study established the functional outcomes of LPS-pretreated exosomes, the underlying molecular mechanisms remain insufficiently explored. For instance, we have not yet identified the specific key molecules (such as particular miRNAs or proteins) carried by these exosomes, nor have we elucidated how they regulate downstream signaling pathways to underlie their synergistic effects.

## Conclusion

Research indicates that controlling oxidative stress and mitochondrial dysfunction is crucial for alleviating periodontal inflammation and promoting tissue regeneration during the progression of periodontitis. This study demonstrates that exosomes derived from LPS-BMSCs are more effective than BMSCs-Exo in ameliorating oxidative stress and mitochondrial dysfunction, inhibiting cellular apoptosis, and significantly promoting osteogenic functional recovery. In a rat periodontitis model, LPS-BMSCs-Exo exhibited superior therapeutic potential, significantly reducing alveolar bone resorption and inflammatory levels. These findings reveal the intrinsic relationship between regulating oxidative stress through mitochondrial function improvement and promoting osteogenic differentiation, providing new perspectives for developing oxidative stress-targeted therapeutic strategies for periodontitis.

## Supplementary Information

Below is the link to the electronic supplementary material.

Supplementary Material 1.

Supplementary Material 2.

## Data Availability

All the data are available in the main text or the supplementary materials.

## Abbreviations

LPS: Lipopolysaccharide
BMSC: Bone marrow mesenchymal stem cell
Exo: Exosome
TEM: Transmission electron microscopy
NTA: Nanoparticle tracking analysis
PDLSCs: Periodontal ligament stem cells
ROS: Reactive oxygen species
SOD: Superoxide dismutase
ATP: Adenosine triphosphate
PBS: Phosphate buffer solution
HE staining: Hematoxylin‒eosin staining
ABC: Alveolar bone crest
CEJ: Cementoenamel junction
BMD: Bone mineral density
BV/TV: Bone volume/total volume
