# Supplementary material for: Exosomes from LPS-pretreated BMSCs treated periodontitis via improving oxidative stress
Source: Stem Cell Res Ther. 2025 Dec 31;17:62. doi: 10.1186/s13287-025-04860-y (PMC12865969; doi:10.1186/s13287-025-04860-y)
Supplement: Supplementary file 1 — Supplementary Material 1. [file 13287_2025_4860_MOESM1_ESM.docx]

**Supplementary File 1**

for

**Exosomes from LPS-pretreated BMSCs Treated Periodontitis**

**via Improving Oxidative Stress**

**Chenyu Xu ^a, b, 1^, Hanping Wang ^a, b, 1^, Wenqi Dong ^a, b^, Wen Cheng ^a, c^, Yuran Su ^a, b^, Qiang Yang ^d, e^, Yue Wang *^,^ ^a, b^, Yanhong Zhao*^,^ ^a, b^**

**^a^ Department of Orthodontics, Tianjin Medical University School and Hospital of Stomatology & Tianjin Key Laboratory of Oral Soft and Hard Tissues Restoration and Regeneration, No.12 Qixiangtai Road, Heping District, Tianjin 300070, P. R. China**

**^b^ Tianjin Medical University Institute of Stomatology, No.12 Qixiangtai Road, Heping District, Tianjin 300070, P. R. China**

**^c^ Department of Stomatology, Liangxiang Hospital of Beijing Fangshan District, Beijing 102400, P. R. China.**

**^d^ Department of Spine Surgery, Tianjin Hospital, Tianjin University, 406 Jiefang South Road, Tianjin 300211, P. R. China**

**^e^ Department of Orthopaedics, Beijing Friendship Hospital, Capital Medical University, 100050, Beijing, China**

***Corresponding author**

**E-mail address: yzhao@tmu.edu.cn (Yanhong Zhao), wangyue1@tmu.edu.cn (Yue Wang)**

**^1^ These authors contributed equally to this work.**

**E-mail address: xuchenyu@tmu.edu.cn (Chenyu Xu), wanghanping@tmu.edu.cn (Hanping Wang)**

Supplemental Figure 1: CCK-8 was used to detect the effect of different concentrations of LPS on the activity of PDLSCs. * * *P* < 0.01; * * * * *P* < 0.0001


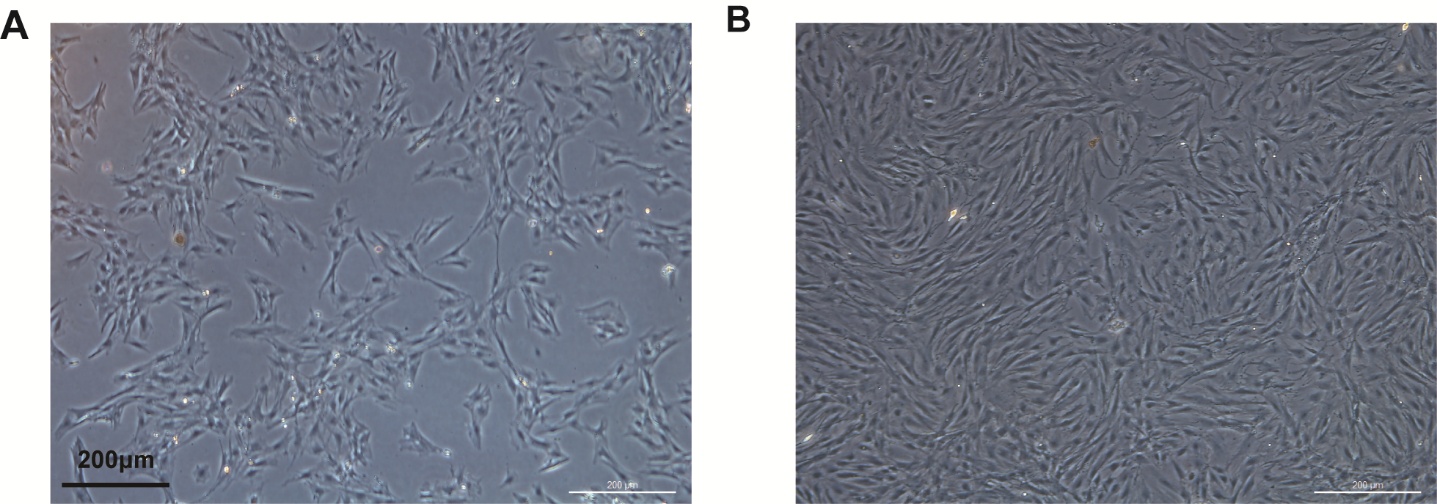


Supplemental Figure 2：The photographs of BMSCs under light microscope, (A) before LPS treatment, (B) after LPS treatment for 48 h.


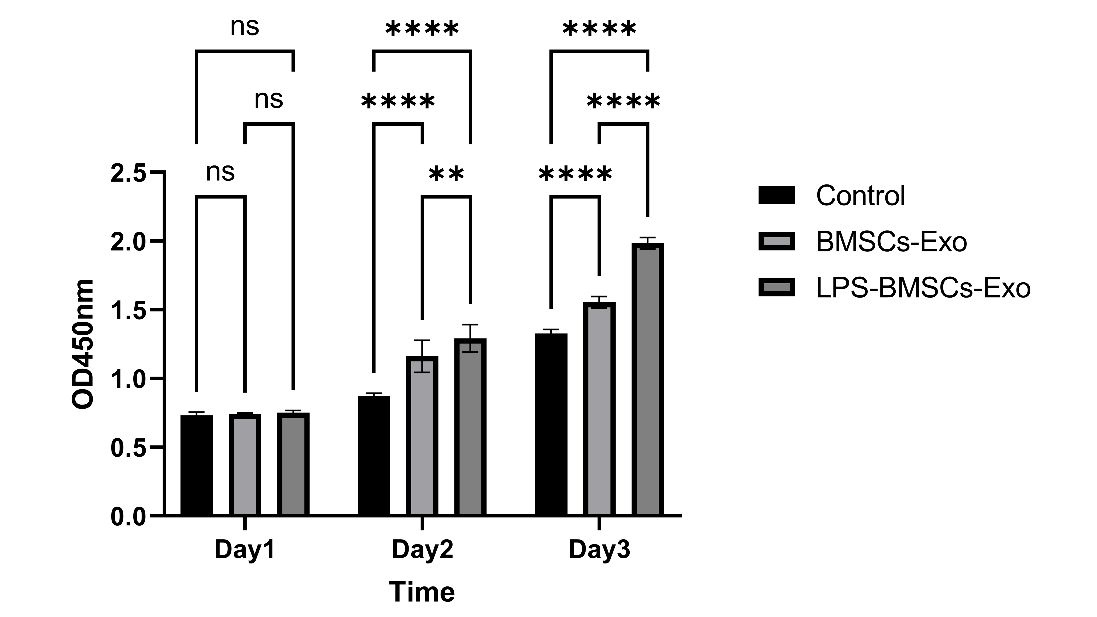


Supplemental Figure 3：CCK-8 was used to detect the effect of different concentrations of LPS on the activity of PDLSCs. ***P* < 0.01; *****P* < 0.0001


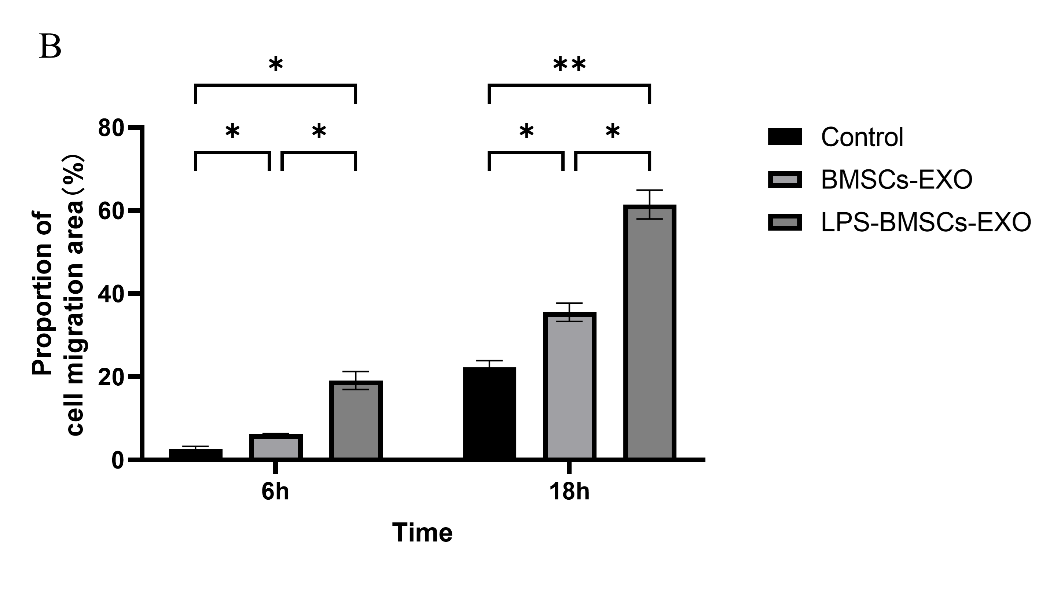


Supplemental Figure 4：The wound healing assay was used to evaluate the effect of different concentrations of LPS on the migration ability of PDLSCs. ***P* < 0.01; **P* < 0.05


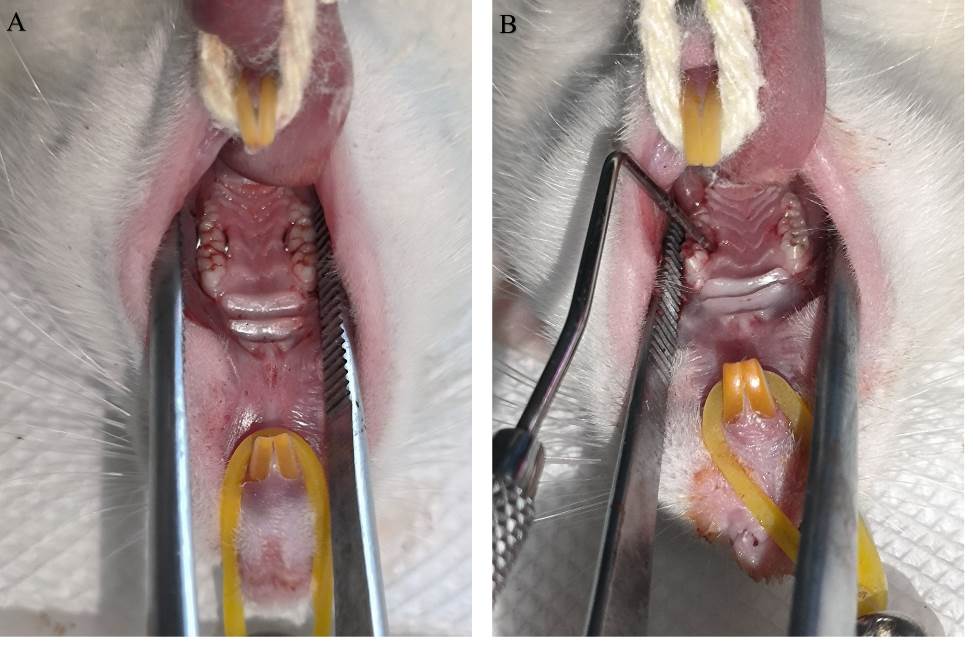


Supplemental Figure 5: Pictures of periodontitis model in rats

1. Ligation of bilateral maxillary second molars in rats (B) Successful modeling of periodontal probing deep bleeding


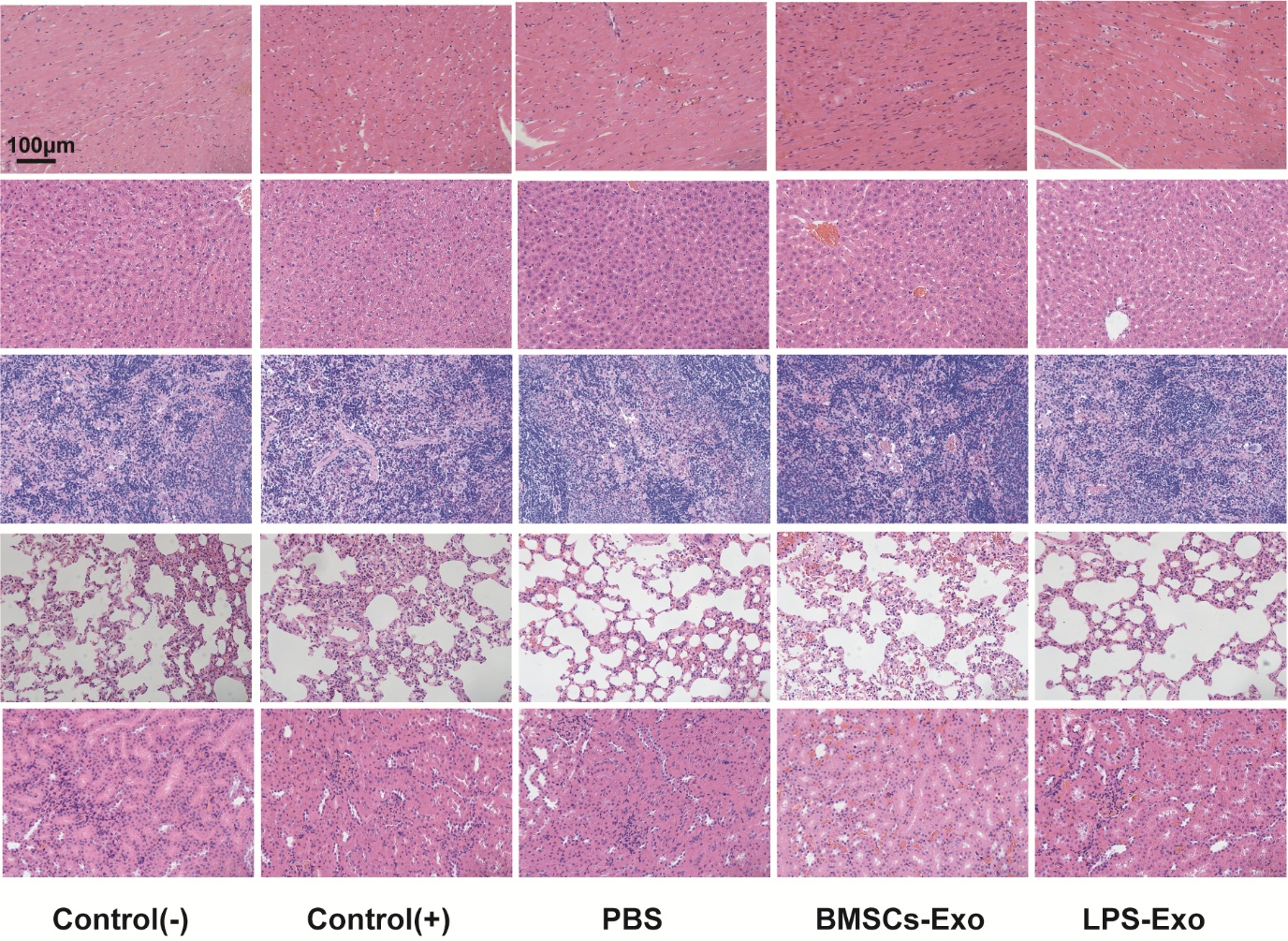


Supplemental Figure 6: HE staining results of organs in each group, scale: 100 μm
