# Supplementary material for: Exosomes from LPS-pretreated BMSCs treated periodontitis via improving oxidative stress
Source: Stem Cell Res Ther. 2025 Dec 31;17:62. doi: 10.1186/s13287-025-04860-y (PMC12865969; doi:10.1186/s13287-025-04860-y)
Supplement: Supplementary file 2 — Supplementary Material 2. [file 13287_2025_4860_MOESM2_ESM.docx]

**Supplementary File 2 (Figures of uncropped blots)**

for

**Exosomes from LPS-pretreated BMSCs Treated Periodontitis**

**via Improving Oxidative Stress**

**Chenyu Xu ^a, b, 1^, Hanping Wang ^a, b, 1^, Wenqi Dong ^a, b^, Wen Cheng ^a, c^, Yuran Su ^a, b^, Qiang Yang ^d, e^, Yue Wang *^,^ ^a, b^, Yanhong Zhao*^,^ ^a, b^**

**^a^ Department of Orthodontics, Tianjin Medical University School and Hospital of Stomatology & Tianjin Key Laboratory of Oral Soft and Hard Tissues Restoration and Regeneration, No.12 Qixiangtai Road, Heping District, Tianjin 300070, P. R. China**

**^b^ Tianjin Medical University Institute of Stomatology, No.12 Qixiangtai Road, Heping District, Tianjin 300070, P. R. China**

**^c^ Department of Stomatology, Liangxiang Hospital of Beijing Fangshan District, Beijing 102400, P. R. China.**

**^d^ Department of Spine Surgery, Tianjin Hospital, Tianjin University, 406 Jiefang South Road, Tianjin 300211, P. R. China**

**^e^ Department of Orthopaedics, Beijing Friendship Hospital, Capital Medical University, 100050, Beijing, China**

***Corresponding author**

**E-mail address: yzhao@tmu.edu.cn (Yanhong Zhao), wangyue1@tmu.edu.cn (Yue Wang)**

**^1^ These authors contributed equally to this work.**

**E-mail address: xuchenyu@tmu.edu.cn (Chenyu Xu), wanghanping@tmu.edu.cn (Hanping Wang)**

1. CD9





Bright field figure


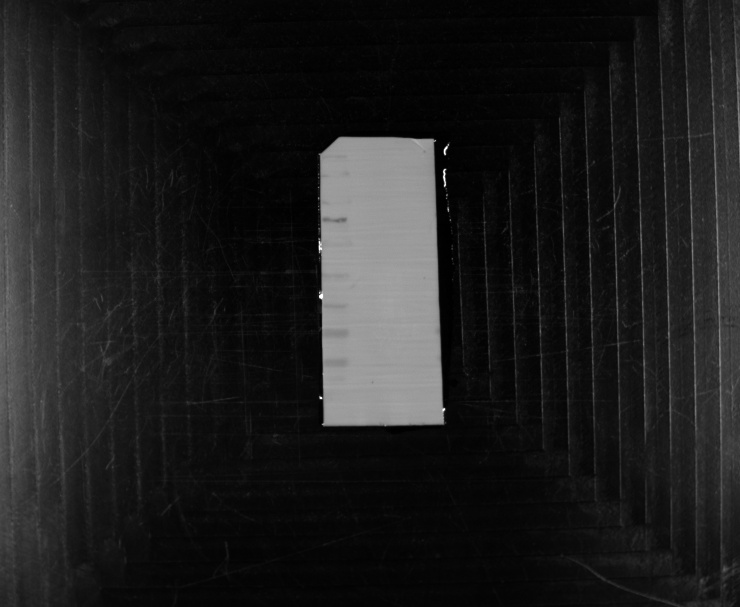


Chemiluminescence figure





Merged figure

B. CD81





Bright field figure


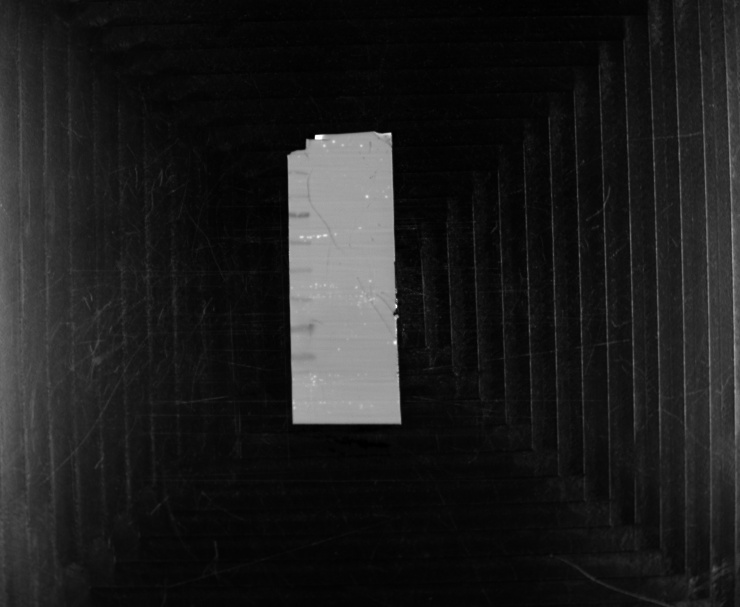


Chemiluminescence figure





Merged figure

C. TSG101





Bright field figure


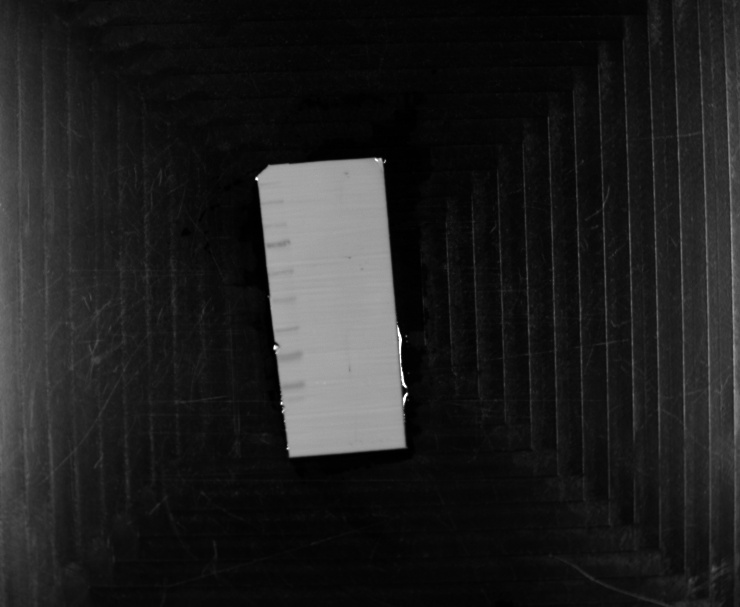


Chemiluminescence figure





Merged figure

D.Galnexin





Bright field figure


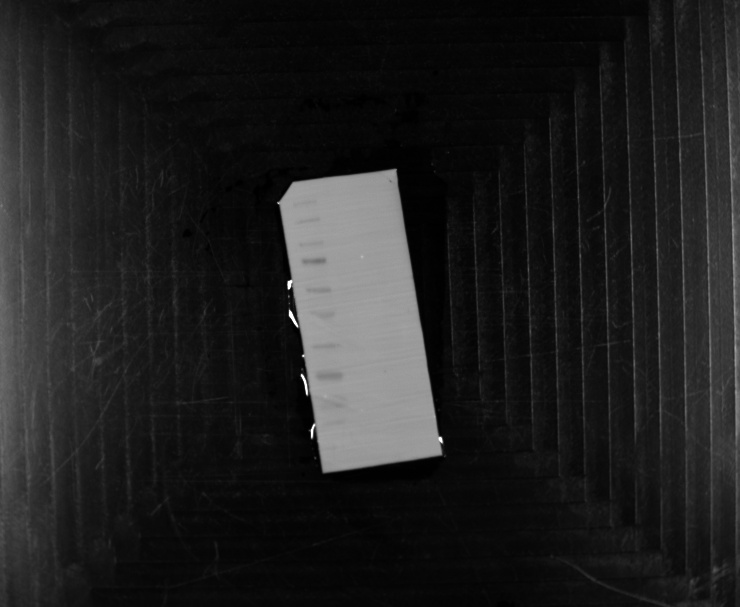


Chemiluminescence figure





Merged figure
